# The Vineyard Microbiome: How Climate and the Main Edaphic Factors Shape Microbial Communities

**DOI:** 10.3390/microorganisms13051092

**Published:** 2025-05-08

**Authors:** Vanessa Silva, Isabel Brito, Ana Alexandre

**Affiliations:** 1 MED-Mediterranean Institute for Agriculture, Environment and Development & CHANGE—Global Change and Sustainability Institute, IIFA-Institute for Advanced Studies and Research, Universidade de Évora, Pólo da Mitra, Ap. 94, 7002-554 Évora, Portugal; d38740@alunos.uevora.pt; 2 MED-Mediterranean Institute for Agriculture, Environment and Development & CHANGE—Global Change and Sustainability Institute, Department of Biology, School of Science and Technology, Universidade de Évora, Pólo da Mitra, Ap. 94, 7002-554 Évora, Portugal; ibrito@uevora.pt

**Keywords:** soil microbiome, abiotic factors, edaphic factors, *terroir*, grapevine, bacteria, fungi

## Abstract

The soil microbiome is a complex system that encompasses millions of microbes including archaea, bacteria, fungi, protozoa and viruses. The role of abiotic factors is crucial in shaping the distribution patterns of microorganisms, its abundance and also the interactions between species, from local to the global level. In the particular case of the vineyard, the microbial communities have a potential impact in both the grapevine development and health and, later on, in the grape production and quality. The present review focuses on how the composition of soil microbial communities is influenced by climate and several edaphic factors, such as soil moisture, soil nutrients and soil pH. It also discusses the role of microorganisms and their metabolic activity on the fermentation process, influencing the sensorial characterisation of the wine and suggesting the definition of a microbial *terroir*.

## 1. Introduction

Soil ecosystems are complex habitats harbouring diverse communities of microorganisms, including bacteria, fungi, archaea, protists and viruses forming interactive networks that sculpts the soil microbiome which impacts both plant and animal health [1].

Soil microorganisms are key drivers in this ecosystem functioning, directly impacting soil fertility and plant productivity [2,3,4]. Plant roots represent a unique ecological niche for soil microorganisms and the root-associated microbiome (both outside and inside the root) known for their important role in plant health and plant protection against abiotic and biotic stresses [5]. The wine *terroir* is a multidimensional concept, which refers to a distinct profile including climate, soil, landscape and cultural practices responsible for the unique traits of wine, also shaped by soil microorganisms and playing a determinant role in viniculture [6]. Soil microbiome significantly impacts the microbial communities of the phyllosphere, which have strong implications for plant resilience, growth, and vigour. Bacteria and fungi are the most studied groups of soil microorganisms; however, other communities as, for example, microalgae, are also important players in soil fertility and plant health [7].

**Figure 1:**
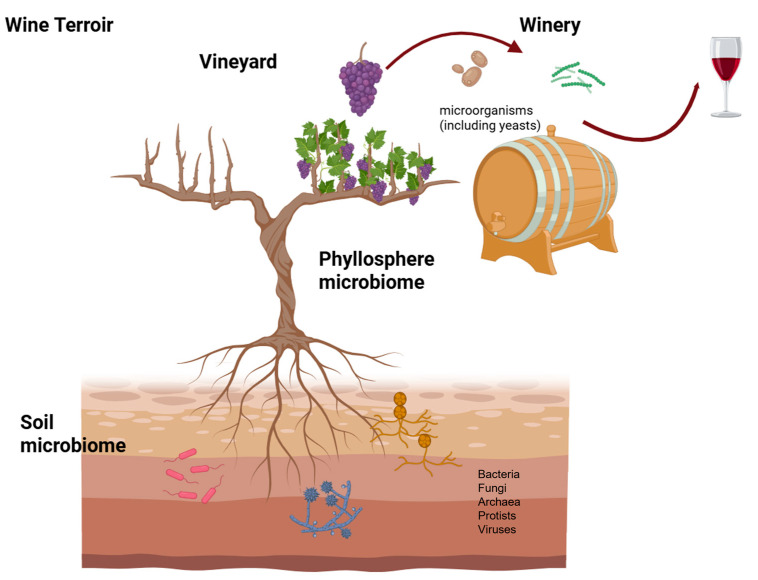
Exploring the vineyard microbiome (created with BioRender.com, accessed on 24 April 2025).

The grapevine (*Vitis vinifera* L.) phyllosphere is characteristic of each region in which some species prevail over others. Hence, together with cultural practices and associated to other soil characteristics as physicochemical properties, the soil microbiome contributes for the unique and distinctive character of wines from a particular region (Figure 1).

The soil-related microbiota has been considered crucial, not only for the biochemical properties of soils but also for grapevine health, yield and wine quality [8]. Bokulich and co-workers [9] have shown that microbial community of wines is shaped by the grape variety, biogeographical and climatic factors. Results from other studies concluded that microbial communities from different geographical locations varied both at the genetic and phenotypic level [10]. According to another study from the same research team [11], there is a strong association involving grapevine microbiota, fermentation characteristics and wine chemical composition, flavour and quality. Exploring the main factors that influence soil microbial communities may elucidate how to improve biological processes, soil quality and crop productivity [12,13,14] and in the particular case of grapevine contribute to a better understanding of the *terroir* [15,16,17]. A number of studies have demonstrated that the diversity and community composition of soil bacteria and fungi are mainly influenced by abiotic factors at different scales, such as soil pH and moisture, soil nutrients and climatic conditions [18,19,20]. In addition, the new border in plant science is the definition of healthy microbiomes for crop production systems to encounter beneficial microorganisms that could contribute to plant protection and productivity in agricultural systems. A significant body of literature has demonstrated the relationship between soil and crop quality, especially with respect to grapes used in wine production. Soil serves as a very important reservoir of vine-associated bacteria, and edaphic factors and vineyard-specific physiological properties can influence the native grapevine microbiome before veraison [21]. The soil microbiota pool impacts grapevine-associated microbiota, which is linked to vine fitness [22].

Under the same climate condition, soil spatial variability highly influences the interactions between the grapevine, soil and the atmosphere [23]. According to Burns and colleagues [11], soil properties, especially those correlated with resource availability to soil microorganisms, such as plant residues or other types of biomass and soil structure, differ in functions of vineyard management. Soil properties affect vine phenology and consequently the composition of grapes that are converted into wine, which may be acknowledged as a specific effect of its regional uniqueness, known as *terroir* expression [24]. Soil microbial activity, which may be evaluated by estimating the activity of specific soil enzymes, is a significant bio-indicator of wine quality because of the major influence of microbes in the wine transformation processes. Importantly, the existence of particular regional microbial biomarkers, able to predict the metabolite composition of the wine, could create a baseline of the regional microbial structure indicating the connectivity between the region and local microbiome. Soil physicochemical properties can also influence the population structure of pathogens, and recently the studies of plant–soil microbiome have addressed the detection of microbial pathogens [25]. The microbial taxa associated to the grape also have a major contribution to pathogen defence and changes the hormonal activity of the plant, thereby affecting its stress tolerance [21].

Since ecosystems depend upon a complex interaction of biological and non-biological factors, addressing each factor separately renders only a partial view of the system. In the last few years, the analysis of soil microbial population dynamics has benefited from the more affordable prices of next-generation sequencing (NGS), and therefore more data from diverse ecosystems are available. The present review focuses on the current state of the art on how abiotic factors shape the bacteria and fungi communities on vineyards and how this may contribute to the *terroir* definition.

## 2. Major Edaphic Factors That Shape Microbial Communities

### 2.1. Geographic Factors

According to the Darwinian concept, microbial ecology relies on the idea that microorganisms are disseminated globally and adapt to proliferate anywhere in any habitat with sufficient environmental conditions. According to the Baas Becking hypothesis, “Everything is everywhere but the environment selects” [26,27]. Nevertheless, some studies support the opposite, i.e., the dispersion limitation may impact microbial communities, which suggests a negative correlation between similarity of microbial assemblages and geographic distance, regardless of the environmental factors. Geographic distance and environmental conditions are considered to be the main factors in the modulation of microbial communities. In fact, the environment is dependent on the geographic location, as it is considered to become different with distance [28]. Recent studies on vineyard microbiome report the existence of a structured microbiota, which depends on the cultivar, geographical factors such as the vineyard mesoclimate, topoclimate and microclimate, soil geology and pedology and the agronomic approach used [11,29,30]. All these factors contribute to the expression of the *terroir* [31,32]. Several studies have already confirmed the influence of geographical location on shaping the *terroir* of grapevines and also the important role of the microbial community, as well as its spatial distribution between and within vineyards, for defining viticultural zones and regional wine properties [8,21,33,34]. Results from a recent study performed in the Hexi Corridor desert in Northern China demonstrated that increasing geographic distance can lead to declining similarities in microbial communities [35]. On the other hand, Hazard and co-workers [36] showed that the distribution of arbuscular mycorrhizal fungi (AMF) at a landscape scale was determined by local environment instead of the geographical distance. In addition, this study found strong evidence of a relationship between environmental factors, mainly soil factors, such as soil available potassium, soil organic carbon (SOC) and available nitrogen in the construction of major fungal communities. Regional factors, such as regional climatic factors and local edaphic variables, were also reported to affect the fungal biodiversity within Californian sub-regions [37] and Portuguese appellations (protected areas legally defined by geo-political boundaries) [38]. Some studies indicate that significant biogeographic patterns are in general detected at large scales, suggesting that the composition of soil microbial communities changes significantly to large spatial extents (e.g., landscapes, regions and continents) due to environmental heterogeneity in factors such as soil texture, climate, plant community composition, availability of labile carbon and soil pH. Other studies report that it is possible to verify biogeographic patterns at multiple spatial scales [39]. Considering smaller geographical scales when comparing microbial communities (at the scale of individual vineyards), the geographical patterns among populations are often more evident for fungi than for bacteria [31]. However, exactly how the set of pervasive species alters from global to local scale and which factors can significantly change its composition remains unclear. If similar general ecological mechanisms are the key drivers of the establishment of bacterial and fungal communities in soil, we could expect that core populations of bacteria and fungi have the same dependency on location and land use [40]. Furthermore, understanding how microbial groups differ in space and time is fundamental for an integrated understanding of ecosystem function [41,42,43,44]. A study addressing small vineyards of the Napa Valley AVA showed that the microbial communities were generally different across locations and influenced by geography and farming practices, similarly to the findings by Burns and colleagues [8], which showed that differences in soil bacterial communities at the landscape scale result from environmental and edaphic factors. This suggests that dispersal constraints contribute to soil bacteria differences at a much smaller scale. Nevertheless, concerning small geographic scales, there are still few studies designated to understanding whether vineyard microbiomes show different patterns of distribution (e.g., comparing neighbouring vineyards) and how such patterns are associated with a wine’s *terroir*. These differences are often correlated with geographic distance between vineyards, considered as a prior factor, which contributes to bacterial community differences at a much smaller scale than previously reported. Additionally, environmental variables (e.g., climatic, topography, soil properties, and management practices) were considered the major drivers to such dissimilarities [45], despite the fact that is not clear whether geographic separation is a merely representative of other drivers [8]. The specific environmental constraint at a local stage is commonly the most important variable in studies of soil microbiome. Conversely, several studies show that there is not enough available data to support the existence of wine *terroir*-specific species and/or strains, and that the reports concluding otherwise are maybe based on short sample numbers, not taking into account the wide diversity of microbial populations in space and time [9,12,38].

According to Bokulich and colleagues [11], geographical delineations of yeast communities have been documented in several wine appellations and the differences were associated to specific wine metabolomes. Consequently, grape microbiota and wine metabolite features delineated viticultural area designations and individual vineyards in Napa and Sonoma Counties, California [27]. With a central role on the wine-making process, yeasts are driven from the surroundings to the berry surface and are expected to develop between the veraison and harvest period, depending on the nutrient availability of berry skin, which in turn is also dependent on the environment influence. According to this line of thought, there is little evidence that the grapevine microbiota may be restricted by or coincident with the regional borders related to the *terroir*. According to the work of Pinto and co-workers [38] that aimed to prove the influence of soil microbiome on wine *terroir* through the characterisation and comparison of the microbial populations during spontaneous wine fermentations, considering several Portuguese appellations, significant differences were observed among the three stages of fermentation, which were analysed in terms of both fungal and bacterial communities. Additionally, this study found a regional effect on the microbial populations in initial musts; nevertheless, bacterial communities were found to be less responsive to fermentation stages or geographical origin than the corresponding fungal populations. The experimental approach of this study [38] revealed the dynamics of microbial communities of spontaneous fermentation presented at the initial must (IM) and the biogeographic distribution of grape and wine microbiomes of six Portuguese wine appellations, which revealed significant differences for both bacterial and fungal communities at the IM for all the wine appellations. These findings proved the existence of geographic influence on wine fermentation microbiome, as different ecological niches, which alter in terms of microbial populations, may have a positive impact on the final wine sensorial properties and consequently increase in wine quality.

Regarding the fungi population, Alentejo appellation was associated to *Lachancea* contributing 21.44% of the region’s similarity; *Rhodotorula* and *Botryotinia* were detected in the Estremadura appellation contributing 37.96% of the similarity; *Ramularia* and *Hanseniaspora* were the major genera in Bairrada with a regional similarity of 18.86%; *Rhodotorula* and *Lachancea* genera were the most prevalent in Dão (29.07% of similarity); the Douro appellation was characterised by the presence of fungi *Rhodotorula* and *Erysiphe* (21.29% of similarity) and finally, for Minho appellation, *Rhodotorula* and *Alternaria* contributed 40% of similarity [29,38,46]. Additionally, *Hanseniaspora uvarum*, *Metschnikowia pulcherrima*, and *Pichia terricola* were the major species in wine-producing regions of the Azores Archipelago in North Atlantic Ocean [47]. Regarding the bacterial community at initial must and for the same six Portuguese appellations mentioned above, *Enterobacteriaceae*, *Pseudomonadaceae*, *Microbacteriaceae* and *Comamonadaceae* families represented 52.68% of the group similarity, followed by *Oxalobacteraceae*, *Sphingomonadaceae*, *Xanthomonadaceae*, *Nocardioidaceae*, *Methylobacteriaceae*, *Halomonadaceae*, *Propionibacteriaceae*, *Rhodobacteraceae*, *Micrococcaceae* and *Acetobacteraceae* with a contribution of 38.25%. Furthermore, the diversity of grape fungal community of *Vitis vinifera* L. cultivars is also affected by farming practices. Vineyards of the Montepulciano cultivar that employed conventional, organic or biodynamic managements showed to harbour different fungal communities. These systems differed mainly in terms of the restricted chemical applications allowed on organic and biodynamic farming compared to conventional farming (both organic and biodynamic types follow specific regulation and certification). In all the previously mentioned soil management practices, the most abundant fungal genera were *Hanseniaspora* (wine fermentation potential), *Areobasidium* (wine fermentation potential) and *Botrytis* (plant pathogen), with relative abundances over 30%, 20% and 10%, respectively [48]. It is noteworthy that some species were more frequent or present only on the organic and biodynamic systems, namely *Areobasidium pullulans*, *Cladosprium cladosporioides*, *Saccharomyces* spp. and *Pseudopithomyces* spp. In addition, some fungal genera associated with spoilage (*Zygosaccharomyces* and *Candida*) were only detected on the grapes from the conventional system. According to another study focusing on merlot wines, spontaneous fermentation (SF) proved to have increased fungal community diversity and decreased bacterial community diversity compared to inoculated fermentation (IF), where commercial *Saccharomyces cerevisiae* was added to the must [49]. *Hanseniaspora*, *Starmerella*, *Alternaria* and *Saccharomyces* were the main fungal genera in SF followed by *Tatumella* and *Ochrobactrum* in much lesser quantities, while *Massilia*, *Nesterenkonia* and *Halomonas* were the leading bacteria in IF. The same study revealed that *Saccharomyces*, *Starmella*, *Alternaria*, *Didymella*, *Erysiphe*, *Monilinia*, *Aspergillus*, *Vishniacozyma* and *Filobasidium* were important fungal taxa during fermentation. On the bacterial side, *Massilia*, *Nesterenkonia*, *Tatumella*, *Halomonas*, *Ochrobactrum*, *Pseudomonas*, *Sphingomo*, *Ralstonia*, *Bacteroides* and *Aliihoeflea* were found to be the most important bacterial taxa during fermentation [49]. Results from a previous study showed different bacterial population and specific chemical profiles for the comparison between organically and conventionally produced wines [50]. Nine of the observed fifteen phyla were found in musts from both fermentation techniques (i.e., *Proteobacteria*, *Cyanobacteria*, *Bacteroidetes*, *Firmicutes*, *Actinobacteria*, *Acidobacteria*, *Spirochaetes*, *Verrucomicrobia* and *Fusobacteria*), although the existence of some phyla depended on the applied fermentation technique, namely the addition of SO_2_. Specifically, *Nitrospirae*, *Planctomycetes* and *Tenericutes* were detected solely in the samples from organically fermented must, whereas *Pedobacter*, *Sphingomonas*, *Janthinobacterium* and *Pseudomonas* were detected only in the conventionally produced wine musts [50].

Many studies performed across the world accounted for the identification of diverse yeast community compositions and abundances and showed that populations differ significantly at local scale. Results from a study comprising 34 vineyards of 4 most important viticultural zones in Greece [27] revealed high inter-regional heterogeneity compared to similar studies. This variability can be derived from the diversity of the landscape structure, which comprises islands, plateau and hilly regions that belong to distinct climatic zones, despite their geographic proximity. According to the study performed by Liu and co-workers [31], fungal communities are not randomly dispersed, showing to have different habitat patterns which depends on the development stage of grapevine. More specifically, grapevine habitats and plant development related to root fungal diversity have shown a biogeographic trend on a scale of 5 km, pointing out extensive local variety according to their host plant. In addition, different studies [21,51,52] indicate that fungal diversity is higher in the below-ground (root zone soil, roots) than the above-ground habitats (flowers, leaves, and grapes), as recently pointed out by Anthony and colleagues [53]. Climate is a fundamental factor to our understanding on how the vegetation properties shift along geographical patterns and how vegetation shifts along altitude gradients and, furthermore, predicting how microbial communities respond to environmental change [26]. The significant role of climate in shaping the geographic pattern of microbial distribution and wine quality has already been demonstrated [31]. In general, the most cool and wet regions have a higher ratio of dominant species and lower soil microbial diversity compared to warmer and drier locations. In the study by Stefanini and co-workers [54], it was reported that samples from a wet vintage evidenced a higher fungal diversity compared to the ones from a dry vintage. Thus, concerns over climate change, with focus on drought and the efficiency of water usage, need to take a broader perspective and also take into account the implications of climate change on soil microbiome.

### 2.2. Soil Water Content

Soil water content (SWC) affects the physiological state of both plants and microorganisms [55]. The SWC and physicochemical properties in the grapevine root have been named the major factors shaping the spatial zoning of the rhizosphere microbiome [8,21,56,57]. In summer, respiration and water fluctuations are greater in wet than in dry soils. Respiration depends more strongly on moisture than on temperature [58]. Furthermore, response to moisture is considered to be more rapid in terms of changes in soil microbial biomass and enzymatic activity compared to other soil properties [59]. In other words, a decrease in soil water availability affects the activity and the size of microbial populations, directly or indirectly [59,60,61]. A large part of the studies performed to date on this subject have concerned the effect of fluctuating water content on the soil microbial communities’ structure and processes, including enzymatic activity [58,60]. However, the effect of the soil being continuously inundated or water saturated on soil properties is also important and has been less frequently studied. Both the higher and the lower water content, compared to the optimal values, can change the structure of the active microbial community [62]. Excessive SWC causes limitations in oxygen diffusion because this process is much slower in water than in air, which in turn reduces the rate of aerobic microbial processes and increases the activity of anaerobic microorganisms. In fact, both wet and dry conditions may influence microbial biomass by creating environments unfavourable for both aerobic Gram+ and Gram- bacteria, as well as mycorrhizal fungi [63].

The SWC is also important when associated to the activity of soil enzymes because it affects many biogeochemical processes and is especially significant concerning hydrolytic enzyme activities, which may include pectinases, chitinases, lipases, cellulases and amylases [64]. The enzyme activities can directly affect plant growth by influencing nutrient availability in the environment [65]. In addition, the production of some enzymes may also activate the defence mechanism of the host plant which leads to the induction of the plant immune system against biotic stresses [65]. Grapevine growing is traditionally carried out in non-irrigated, extensive agricultural areas in semi-arid regions and dry lands, which means it has high drought tolerance. Once established in a deep soil with deep root penetration, the grapevines are able to survive extreme water deficits which has significant effects on grape berry composition that consequently can impact wine quality by the intensification of colour, flavours or aroma [66]. Nevertheless, drought conditions reduce the rate of growing and photosynthesis of the grapevine, thus limiting leaf functions. Wine grape yield is largely influenced by the availability of water for growth between flowering and veraison. Despite being a moderately drought-resistant species, *V. vinifera* is affected by soil moisture during the growing season [67], which corresponds to the rapid berry enlargement. Regulated deficit irrigation has been used to enhance berry and wine quality [66]. According to Shellie and Brown [68], the wine grape nutrient status is optimised under a water-deficit status based on petiole sampling at veraison. Soil moisture significantly influences wine quality by affecting vine growth, grape composition and ultimately the flavour of the wine. Different soil types and their moisture levels impact how vines absorb water and nutrients, which in turn influences the development of grapes and their flavour profile. Nevertheless, further studies are required to enhance the current knowledge on the influence of soil water content on the microbial communities’ dynamics and how this influences grape quality and ultimately wine characteristics.

### 2.3. Soil Elements

Soil nutrients are essential for plant growth and health. The fertility of a particular soil is primarily measured by the availability of nitrogen (N), carbon (C), potassium (K) and phosphorus (P) [69,70]. Additionally, micronutrients such as iron, manganese, zinc, copper, boron and molybdenum are essential to the vineyard and may also affect the microbiome. The nutrient status of soil may be very heterogeneous in its spatial distribution, and according to Leibig’s law of the minimum, despite the existing amounts of certain nutrients, neither the nutritional balance nor yield are good if the soil lacks a single mandatory nutrient.

Among mineral elements taken up from the soil by plants, nitrogen is definitely the element that most influences vine vigour, yield, grape ripening and berry quality [71,72,73]. Nitrogen-fixing organisms, namely diazotrophic bacteria and cyanobacteria, contribute to the nitrogen pool in vineyards through the biological conversion of atmospheric nitrogen to ammonia, which is the biological available form to be absorbed by the plant roots. Recent studies have explored the potential of using both diazotrophic bacteria and microalgae as soil biofertiliser (for review, see [74]). While promoting N efficient supply is certainly good for crop productivity, the application of large amounts of N fertiliser during the last few years has caused considerable consequences to the environment, polluting waterways and the coastal zone [75].

Increasing restrictions on the use of nitrogen fertilisation aim to minimise ground water pollution. The European Commission expects a reduction in nutrient losses from fertilisers of at least 50% by 2030 (https://agriculture.ec.europa.eu/sustainability/environmental-sustainability/low-input-farming/nutrients_en, accessed on 23 January 2025). Although the grapevine requires smaller amounts of nitrogen fertilisation than most crops (30 kg/ha vs. 100–200 kg/ha), in some cases it can be a limiting factor causing a major problem to viticulture (for instance, in steeper slopes). Cover crops are used in vineyards for many different purposes as, for example, to prevent soil erosion, improve soil structure, reduce the use of herbicides, increase trafficability during wet weather and/or provide habitat for beneficial organisms [72]. Nevertheless, the existence of a cover crop in the inter-row of a vineyard triggers competition for soil resources, particularly for nitrogen [72,76]. Availability of nitrogen is not a static pool between sites, which alters continually through mineralisation, plant uptake and immobilisation. The decomposition of cover crops’ straw on the soil surface can enhance the availability of nutrients, favouring their absorption by the grapevine. The microbial activity, as influenced by their interactions with plants and other soil organisms, impacts the form and quantity of nutrients that are absorbed by plants. Once organic N is mineralised, plants can effectively compete for ammonia and nitrate uptake. Since plants host microbes and maintain a strong net N flow from soil to roots, they may also compete with microbes in the long run. Especially nitrates (N-NO_3_) can vary rapidly, depending on the mineralisation, nitrification and immobilisation rates that produce and consume new available nitrogen [75,77,78]. There is a considerable lack of studies pointing out the fact that vine N uptake depends mainly on soil parameters such as soil organic matter content, C/N ratio of soil organic matter and organic matter turnover [72]. Van Leeuwen and Rességuier [79] have discussed, based on several studies, that water and nitrogen supply to the vineyards together with soil temperature are the most important soil physicochemical factors responsible for the *terroir* effect. Soils inducing low vine vigour, caused by a lack of nitrogen availability for the vines or low water content, increases fruit zone exposure to light penetration, which is crucial for growing high-quality fruit [72]. A large number of vineyard soils on the Iberian Peninsula are calcareous, and humus on such soils is stabilised by active lime and is difficult for bacteria to mineralise (degrade). The annual rate of mineralisation is much lower than that of other vineyard soils. For this reason, this type of vineyard soil requires excessive amounts of nitrogen in order to guarantee a balanced nitrogen supply [80]. The importance of soil nitrogen availability in microbiome richness and plant productivity has been demonstrated; however, it was to the detriment of lower plant and bacterial community abundance and diversity [81]. Moreover, the study performed in [82] has shown that N addition significantly decreased soil bacteria diversity and yet had no relevant effect on soil fungal diversity and microbial (bacterial or fungal) richness. An effective controlled N application on vines avoids excessive vegetative growth and should be able to erase adequate yeast in the grapefruit and consequently promote the synthesis of flavour and aroma compounds (mainly in white wines) [83]. In addition, low nitrogen can stimulate polyphenol synthesis, while an excess can originate diluted flavours and excessive vine growth [79].

The relative proportion of fungal and bacterial biomass in the soil can be reflected on the microbial carbon-to-nitrogen ratio (C:N) ratios, as higher ratios tend to indicate more fungi present in the soil than bacteria [84]. The C:N ratio is a major factor that determines the speed of organic material decomposition as well as nutrient release patterns. Low C:N ratio favours fast decomposition, resulting in quick release of nutrients. Many beneficial organisms responsible for decomposition can multiply fast and obtain their food by decomposing materials with a low C:N ratio [85]. According to Sun and colleagues [86], carbon also determines the structure and function of microbial communities in the soil. Despite the existence autotrophic microorganisms in soil, the major part is heterotrophic, consuming organic carbon for their potential growth through the mineralisation of organic composts. Therefore, there is a very strong relationship between carbon composition and soil microbial community structure and functionality. In addition, soil microbial community composition and microbial activity depended on the type of organic amendment added to the soil [87]. These findings are corroborated by the study performed by Liu and co-workers [31] in conifers trees, pointing out the complexity of the relationship between total soil organic carbon (TOC), total nitrogen (TN), soil water content (SWC), soil microbial community composition and soil functions. The carbon originating in the grapevine root, together with soil chemical and physical properties such as moisture, texture and pH together with the different agricultural practices, set the environment for its rhizosphere microorganisms [88]. Consequently, changes in leaf and grape microbiota can also be associated to soil carbon content and chemical forms and showed interannual variations [21]. 

Phosphorus (P) is essential for plant growth and soil microbial activity. Therefore, the concentration of P in soil is much higher than in the plant due to the multiple forms of P, aluminium/iron or calcium/magnesium phosphates existing in the soil, which limit their availability for plant uptake. Consequently, it provokes a decrease in photosynthesis activity, which affects crop yield and quality. This nutrient is of major importance in the synthesis of biomolecules and formation of high-energy molecules, cell division, enzymatic activity and carbohydrate metabolism. The application of this nutrient in the soil was seen to change the structure of bacterial and fungal communities, with a generalised positive effect on soil microbial abundance [89]. In grapevines, P plays a vital role, primarily through its impact on grape berry composition and vine growth, with repercussions in the must and the final wine. At the same time, P limitation can originate poor vegetative and reproductive growth, namely stunted shoots, affecting grape yields and berry quality. On the other hand, excessive phosphorus rates can also negatively impact grape quality, sugar content decrease and other undesirable compounds [90].

Potassium (K) is an is an essential plant nutrient of major importance in plant physiological and metabolic processes, such as photosynthetic carbon assimilation. It also develops plant drought resistance and protects against oxidative stress and diseases. A recent study reported that potassium application can reduce microbial diversity (both bacterial and fungi) as it may favour particular plant-beneficial microbe groups [91]. Potassium levels are of relevant interest for winegrowers because they influence grapevine growth, berry composition, as well as the fermenting must and wine quality [92].

Micronutrients, such as iron, zinc, manganese and molybdenum, are the keystone in the structure and function of soil microbiomes. They affect microbial abundance, diversity and network connectivity. In the study performed by Dai and colleagues [93], the relationship between metallic micronutrients and the soil microbiome is well demonstrated. This study indicates that bacterial populations are more influenced by micronutrients than fungal populations. The most influent elements are Fe, followed by Mn, Cu and Zn, which help the improvement of ecosystem productivity, both directly, through the micronutrient accessibility to plants, and, to a minor extent, indirectly, impacting the microbiome.

The comprehensive understanding of the link between soil microbiome and grapevine nutrient uptake requires more studies focusing on the different soil microbial functional groups and their role on nutrient cycling, considering time and spatial variations. Moreover, cover crops can increase the abundance and diversity of beneficial soil microorganisms and thus influencing the balance of nutrients available to the grapevine.

### 2.4. Soil pH

Soil pH is one of the most influential variables in soil and is a powerful factor impacting the size, activity and community structure of the soil microbial populations [94,95]. In fact, soil pH is thought to outcome the influence of spatial and climatic factors (biomes) and is also reported to be more important than nutrient content for shaping bacterial communities in agricultural soil in terms of ecological function and biogeographic distribution [95,96]. Soil pH can significantly affect vineyard productivity and grape quality. Despite the fact that vines are considered to be resilient to different growing conditions, soil pH should be controlled in order to secure that the nutrients are available to the plant.

This parameter strongly influences other abiotic factors, such as carbon and nitrogen dynamics, as it significantly alters soil microbial activity and rates of soil C and N cycling [97]. Nevertheless, the difficulty in predicting the influence of pH on the composition of the microbiome has to do with the difficulty in determining whether bacterial and/or fungal communities are directly or indirectly influenced by pH. Some studies show that soil pH has a much more significant effect on bacterial composition and diversity compared to fungal communities, which is probably due to the narrow pH ranges for optimal growth of bacteria, while fungi generally exhibit wider pH ranges for optimal growth [94,98]. On the other hand, the influence of soil pH may be due to the alteration of soil characteristics (e.g., nutrient availability) and thus be an imposed physiological constraint [99].

Considering grapevine nutrition, “strongly acidic” soil is generally of a pH of 5.5 or lower and may cause nutrient imbalances, more often found in nitrogen, phosphorous, potassium, calcium and molybdenum. In contrast, high pH reduces nutrient availability for plant absorption. “Slightly acidic” (pH of 5.6–6.9) and neutral (pH of 7.0) soils have better nutrient balance and are most suitable for grapevines, since roots can acquire nutrients and vineyards can grow to its potential [100].

Soil pH explains the distribution of at least 75% of global bacterial genera, which differs across pH values, increasing from acidic levels to neutral across all biomes. Whang and colleagues [96] reported that the relative abundance of the dominant phylum, Actinobacteria, decreased with decreasing pH; on the other hand, Proteobacteria and Acidobacteria presence was seen to increase with decreasing soil pH. Genera with acidic optima, and also fungi, are more prevalent in humid climates, such as tropical forests, arctic tundra, and boreal forests, whereas genera with alkaline optima are generally dominant in arid grasslands and drylands [98]. According to some studies that demonstrated threshold effects of microbial occurrence, neutral or slightly alkaline conditions favoured bacterial growth, while acid pH favoured fungal growth [94]. In addition, an optimal soil pH condition for bacterial and fungal diversity in acidic and alkaline soils has been shown at values of ~5.5 and ~8.3, respectively [101]. Growth measurement data indicated that the bacterial growth decreased and the fungal growth increased with lower pH, and in the case of forest soils, the fungal growth rates were higher with pH values below 4. Nevertheless, between pH 4 and 4.5, a significant decrease occurred in the growth rate and biomass, which suggests that other factors may be affecting the total microbial population, such as aluminium (Al) and related toxicity, that directly affects the plant-derived C. In general, a very low pH hinders membrane exchanges and does not favour metabolic processes. Overall, the small change in total activity during the shift between the contributions of fungi and bacteria to soil C mineralisation maybe suggests the complementarity of the two major decomposer groups, indicating their functional redundancy in this process [94]. Collectively, these findings undermine the dynamics of pH effect on both the microbial and physiological aspects of grapevine health and quality.

The study performed by Zahid and co-workers [102] demonstrated the impact of soil properties on grapevine-associated microbiome under the stressful cultivation root-zone restriction during all phenological stages between treatment and control. Among the biochemical parameters analysed, pH was the most statistically significant at the pre-veraison stage, between treatment and control. These findings were corroborated in [21], asserting that soil- and root-associated microorganisms were significantly impacted by soil pH.

## 3. Conclusions

The interactions between soil microbial communities shape the nutrient cycles, which are crucial for any plant growth. Soil microbiome management can contribute to improve agricultural production by increasing crop production and crop protection, enhancing yields and rendering protection against abiotic and biotic stresses and thus contributing to more sustainable agronomic practices. In the case of the grapevine, microbiome influence stands beyond plant growth and plant protection, as it also contributes to grape characteristics and ultimately to wine *terroir* definition. However, it is necessary to understand the time and space dynamics of soil microbiome through deep investigation of vine-associated microbial interactions as well as the drivers of soil microbial community structures and distribution in response to abiotic factors. Despite the increasing number of publications on this area, further studies considering different types of soil as well as different crops and climatic conditions are needed in order to improve the current knowledge on how different factors and their combinations affect vineyards and grape quality. Better understanding the response of soil microbial communities to different conditions will help to manage soil microbiota in order to maximise the benefits of its activity. The development of more sustainable agronomic practices, focused on improving grape production and quality, should consider both soil and phyllosphere microbiome as an ally. The study of the soil microbiome is complex and many aspects remain to be elucidated. Further studies should be developed to better understand how soil microbiome potential can be capitalised in the *terroir*.

Relevant investigation has been conducted on both bacteria and fungi species that could be used as inoculum and benefit the grapevine (for review, see [103]). However, many of these studies are performed under controlled conditions and the transition to field conditions may produce less favourable effects due to competition with the existing microbiome. Nevertheless, these strain-specific studies are paramount to better understand which groups of microorganisms should be considered as more relevant in defining the services provided by the soil and phyllosphere microbiomes. Biofertilisers and biostimulants can positively impact microbial diversity in vineyards once they contain beneficial microbes, able to increase the relative abundance of beneficial bacteria, while biostimulants can contribute to plant growth and stress tolerance that indirectly influences the soil microbiome. However, these products should be used thoughtfully as their indiscriminate use can enclose risks.

## Data Availability

No new data were created or analyzed in this study.
